# NSun2 delays replicative senescence by repressing p27 (KIP1) translation and elevating CDK1 translation

**DOI:** 10.18632/aging.100860

**Published:** 2015-12-14

**Authors:** Hao Tang, Xiuqin Fan, Junyue Xing, Zhenyun Liu, Bin Jiang, Yali Dou, Myriam Gorospe, Wengong Wang

**Affiliations:** ^1^ Department of Biochemistry and Molecular Biology, Beijing Key Laboratory of Protein Posttranslational Modifications and Cell Function, School of Basic Medical Sciences, Peking University Health Science Center, Beijing 100191,China; ^2^ Department of Pathology and Biological Chemistry, University of Michigan, MSI 5215A, Ann Arbor, MI 48105, USA; ^3^ Laboratory of Genetics, National Institute on Aging, National Institutes of Health, Baltimore, MD 21224, USA

**Keywords:** NSun2, p27KIP1, mRNA methylation, translational regulation, replicative senescence

## Abstract

A rise in the levels of the cyclin-dependent kinase (CDK) inhibitor p27^KIP1^ is important for the growth arrest of senescent cells, but the mechanisms responsible for this increase are poorly understood. Here, we show that the tRNA methyltransferase NSun2 represses the expression of p27 in replicative senescence. NSun2 methylated the 5′-untranslated region (UTR) of *p27* mRNA at cytosine C64 *in vitro* and in cells, thereby repressing the translation of p27. During replicative senescence, increased p27 protein levels were accompanied by decreased NSun2 protein levels. Knockdown of NSun2 in human diploid fibroblasts (HDFs) elevated p27 levels and reduced the expression of CDK1 (encoded by *CDK1* mRNA, a previously reported target of NSun2), which in turn further repressed cell proliferation and accelerated replicative senescence, while overexpression of NSun2 exerted the opposite effect. Ectopic overexpression of the *p27* 5′UTR fragment rescued the effect of NSun2 overexpression in lowering p27, increasing CDK1, promoting cell proliferation, and delaying replicative senescence. Our findings indicate that NSun2-mediated mRNA methylation regulates p27 and CDK1 levels during replicative senescence.

## INTRODUCTION

Replicative senescence of cells, a hallmark of aging, is a state of indefinite growth cessation triggered by the replicative exhaustion of cells and sometimes by cellular damage and oncogenic signaling [1]. Expression of p27^KIP1^, a pivotal CDK inhibitor and a tight modulator of CDK-dependent phenotypes, increases with cellular senescence and critically promotes cell growth arrest in replicative senescence [2–4].

Although the role of p27KIP1 in replicative senescence has been well established, its regulation is complex and is elicited at multiple levels. The transcriptional factors FoxM1, FOXO1, and FOXO3a are responsible for the transcriptional control of p27 expression [5–6]. However, the fact that *p27* mRNA levels are largely unaltered during replicative senescence suggests that the rise in p27 expression in senescent cells may be achieved mainly by regulation at the levels of translation or protein degradation [7]. Indeed, regulation of p27 by ubiquitin-mediated proteolysis, a result of phosphorylation at serine 10 (S10) and threonine 198 (T198), has been intensively reported [8–11]. Other studies have also linked the ubiquitin-mediated proteolysis of p27 to the accumulation of p27 protein in cellular senescence [12–14]. In addition to protein degradation, regulation at the level of mRNA translation is also critical for the expression of p27. For example, RNA-binding proteins HuR and CUGBP1 have shown to represses the translation of p27 by interacting with the internal ribosome entry site (IRES) element located at the *p27* 5′UTR [15–16]. Finally, a variety of microRNAs are also involved in the silencing of p27 translation in cancer [17–18].

RNA methylation critically modulates the efficiency and accuracy of translation [19–20], RNA stability [21–22], and the biogenesis of small RNAs [21, 23–24]. NSun2 (NOP2/Sun domain family, member 2; MYC-induced SUN domain–containing protein, Misu) mediates MYC-induced cell proliferation. The expression of NSun2 varies throughout the cell cycle, displaying lowest levels during G1 and highest during S phase [25]. Our previous studies revealed that NSun2 methylates the 3′UTRs of mRNAs encoding p16, p53, E2F3, and ErbB2, thereby enhancing the expression of these proteins in cells exposed to oxidative stress [26–27]. NSun2-mediated mRNA methylation could also promote cell proliferation by elevating the translation of CDK1 [28]. However, the role of NSun2 in replicative senescence and the mechanisms underlying this process have not been studied.

In the present study, we report our findings on the role of NSun2-mediated mRNA methylation in replicative senescence. We describe evidence that NSun2 methylates *p27* mRNA at the 5′UTR. Residue C64 located in the *p27* 5′UTR was identified as a major methylation site. Methylation by NSun2 repressed the translation of *p27* and *CDK1* mRNAs, thereby delaying the process of replicative senescence. Our results high-light the profound impact of NSun2-mediated mRNA methylation on replicative senescence.

## RESULTS

### NSun2 represses p27 translation by methylating the 5′UTR of *p27* mRNA

**Figure 1:**
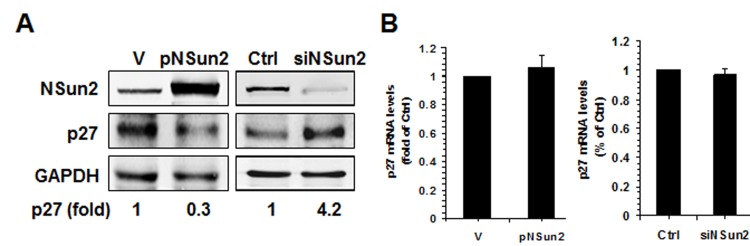
NSun2 regulates p27 expression **(A)** HeLa cells were transfected with a vector expressing NSun2 (pNSun2) or with a siRNA targeting NSun2. Forty-eight hours later, cell lysates were prepared and subjected to Western blot analysis to assess the levels of proteins NSun2, p27, and GAPDH. **(B)** RNA prepared from cells described in Fig. 1A was used for RT-qPCR analysis to assess the levels of *p27* mRNA. Data represent the means ± SD from 3 independent experiments.

**Figure 2:**
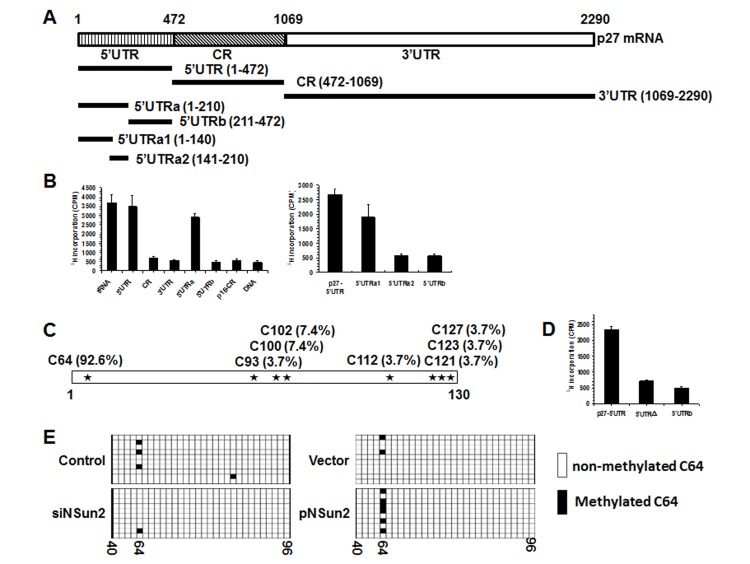
NSun2 methylates *p27* 5′UTR in vitro and in cells **(A)** Schematic representation depicting the *p27* mRNA fragments used for *in vitro* methylation assays.** (B)** Incorporation of ^3^H-labeled SAM into *p27* 5′UTR, CR, 3′UTR, 5′UTRa, and 5′UTRb fragments (*left*) as well as 5′UTR, 5′UTRa1, 5′UTRa2, and 5′UTRb fragments (*right*). The incorporation of ^3^H-labeled SAM into p27 cDNA (DNA) and p16-CR (coding region) served as negative controls. The incorporation of ^3^H-labeled SAM into bacteria tRNA served as a positive control. **(C)** *In vitro* methylated 5′UTRa1 fragment was subjected to bisulfate RNA sequencing analysis to identify the methylation sites, as described in “Materials and Methods”. The percent of methylation at different sites is indicated. **(D)** Incorporation of ^3^H-labeled SAM into the 5′UTR variant mutating C64 (5′UTRΔ). 5′UTR and 5′UTRb served as negative and positive controls, respectively. **(E)** HeLa cells were transfected with a pGL3-derived reporter bearing the p27 5′UTR; 24 h later, cells were further transfected with a vector expressing NSun2 or with NSun2 siRNA and cultured for an additional 48 h. RNA was isolated and subjected to bisulfate sequencing analysis to assess the rate of C64 methylation. Open boxes indicate cytosine-to-uracil conversion, read as thymidine in the cDNA (unmethylated), and filled boxes indicate a retained cytosine (methylated). The numbers below the columns refer to cytosine positions in the *p27* 5′UTR.

The present study was prompted by our earlier findings that high levels of NSun2 lowered p27 abundance. As shown in Fig. 1A, overexpression of NSun2 in HeLa cells by transfection of plasmid pNSun2 reduced p27 protein levels by ~70% while silencing of NSun2 by transfection of small interfering (si) RNA increased p27 protein levels by ~4.2 fold. However, neither overexpression nor knockdown of NSun2 influenced *p27* mRNA levels (Fig. 1B). These results suggested that the regulation of p27 by NSun2 may not involve altered *p27* mRNA transcription or turnover. Because NSun2 had been shown to regulate the expression of p53, p16, E2F3, Bak1, ErbB2, and CDK1 by methylating the mRNAs encoding these proteins [26–28], we tested if NSun2 was capable of methylating *p27* mRNA. To this end, the *p27* mRNA fragments described in Fig. 2A were used for i*n vitro* methylation assays (Materials and Methods). The p27 cDNA and p16-CR (coding region of *p16* mRNA) were included as negative controls, while bacterial tRNA served as a positive control. As shown in Fig. 2B (*left* and *right*), tRNA, 5′UTR, 5′UTRa, and 5′UTRa1 were methylated, while p27 cDNA, p16-CR, p27-CR, p27-3′UTR, p27-5′UTRa2, and p27-5′UTRb were not methylated. Accordingly, the methylation site was located at the *p27* 5′UTR (positions 1-140).

To determine the formation of m5C or m6A in the methylated fragments, *p27-5′UTRa1* methylated *in vitro* by using nonisotopic S-Adenosyl methionine (SAM) and NSun2, and unmethylated (same reaction without adding NSun2) *p27-5′UTRa1* were subjected to MS-HPLC analysis. As shown, m5C was detected in the methylated *p27-5′UTRa1* fragment (Supplemental Fig. S1). Identification of m6A from the methylated *p27-5′UTRa1* did not show any positive results (not shown). To further identify the methylation site by NSun2, *in vitro* methylated *p27-5′UTRa1* was subjected to bisulfate sequencing analysis. As shown in Fig. 2C, among all of the clones sequenced, C64 was detected in ~92.6% of positive clones. Mutation of C64 (p27 5′UTRΔ) nearly abolished the effect of NSun2 on methylating the *p27* 5′UTR (Fig. 2D), indicating that C64 is a major methylation site.

To test whether NSun2 was capable of methylating *p27* 5′UTR in cells, HeLa cells were transfected with a pGL3-derived reporter bearing the *p27* 5′UTR. Twenty-four hours later, cells were further transfected with a vector expressing NSun2 or with NSun2 siRNA and cultured for an additional 48 h. RNA was isolated and subjected to bisulfate sequencing analysis to assess the rate of C64 methylation. As shown, knockdown of NSun2 reduced the methylation of C64, whereas overexpression of NSun2 increased the rate of C64 methylation (Fig. 2E). These results indicate that NSun2 was able to methylate *p27* mRNA in cells.

**Figure 3:**
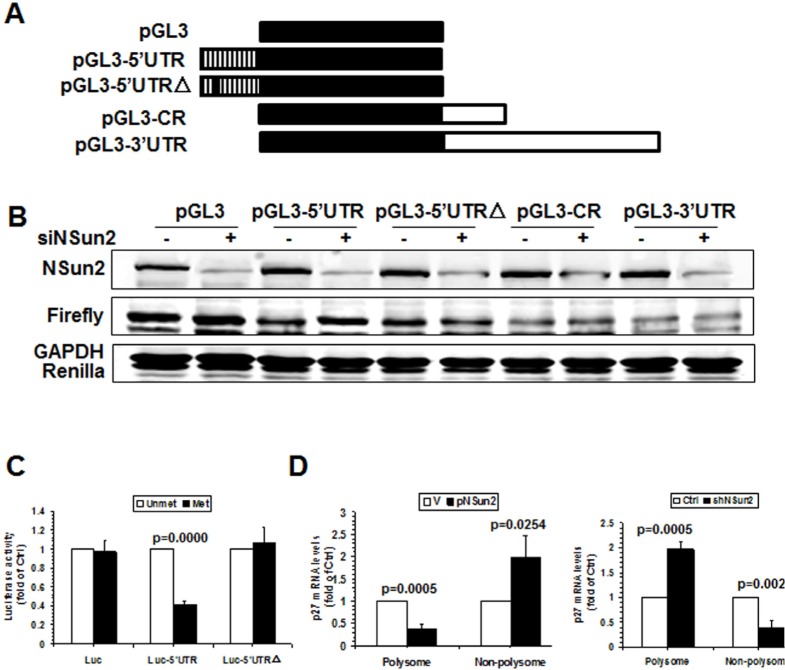
Methylation by NSun2 is functional for regulating the translation of p27 **(A)** Schematic representation of the pGL3-derived reporter vectors used for reporter gene assays. **(B)** HeLa cells were transfected with each of the reporter vectors described in Fig. 3A together with a pRL-CMV control reporter. Twenty-four hours later, cells were further transfected with NSun2 siRNA and cultured for an additional 48 h. Firefly luciferase activity against Renilla luciferase activity was analyzed. Data represent the means ± SD from 3 independent experiments; significance was analyzed by Student's *t* test. **(C)** *In vitro* methylated (Met) or unmethylated (Unmet) Luciferase (Luc), luc-5′UTR, and luc-5′UTRΔ reporter transcripts were used for *in vitro* translation assays. Firefly luciferase activity was measured to determine the translation efficiency. Data represent the means ± SD from 3 independent experiments; significance was analyzed by Student's *t* test. **(D)** Cells described in Fig. 1A were used for isolating the polysomal and non-polysomal fractions. RNA prepared from the fractions was subjected to RT-qPCR analysis to assess the presence of *p27* mRNA in the polysomal and non-polysomal fractions. Data represent the means ± SD from 3 independent experiments; significance was analyzed by Student's *t* test.

To further test whether methylation of *p27* 5′UTR by NSun2 influenced p27 expression levels, pGL3-derived reporters bearing fragments of *p27* mRNA were constructed (Fig. 3A, schematic). HeLa cells were transfected with each of these reporters and 24 h later, cells were transfected with control or NSun2-directed siRNAs and cultured for an additional 48 h. Cellular lysates then were collected and subjected to Western blot analysis to assess NSun2, Firefly luciferase, Renilla luciferase, and GAPDH protein levels. As shown in Fig. 3B, knockdown of NSun2 elevated the levels of reporter protein expressed from pGL3-5′UTR, but not that expressed from pGL3, pGL3-CR, pGL3-3′UTR, or pGL3-5′UTRΔ, indicating that methylation by NSun2 at C64 selectively repressed the expression of p27. Because NSun2 regulates the expression of p27 without influencing the levels of *p27* mRNA (Fig. 1), we further asked if methylation by NSun2 regulated the translation of p27. To this end,* in vitro*-transcribed reporter transcripts Luciferase (Luc), Luc-5′UTR, and Luc-5′UTRΔ were methylated *in vitro* by NSun2 or kept unmethylated. These transcripts then were used for *in vitro* translation assays and reporter activity was used as readout of the efficiency of translation. As shown in Fig. 3C (*left*), methylation by NSun2 reduced the reporter activity of Luc-5′UTR (by ~59%), but not that of Luc and Luc-5′UTRΔ. Therefore, methylation of *p27* 5′UTR by NSun2 represses the expression of p27 at the level of translation.

To further investigate the mechanisms underlying the translational regulation of p27 by NSun2, the polysomal presence of *p27* mRNA in cells with overexpressed or silenced NSun2 was examined. As shown in Fig. 3D, overexpression of NSun2 decreased the presence of *p27* mRNA in the polysomal fraction (by ~66%, p=0.0005) but increased it in the non-polysomal fraction (by ~2.0 fold, p=0.0254). On the other hand, knockdown of NSun2 increased the presence of *p27* mRNA in the polysomal fraction (by ~60%, p=0.0005) but decreased it in the non-polysomal fraction (by ~2.0 fold, p=0.0024). Taken together, these findings indicate that the assembly of *p27* mRNA in the polysome was repressed by NSun2-mediated methylation, in turn repressing the translation of p27.

By binding to the *p27* 5′UTR, RNA-binding proteins HuR and CUGBP1 repress p27 translation [15–16]. These findings raised the question that HuR or CUGBP1 might influence the function of NSun2 in repressing the translation of p27. As shown in Supplemental Fig. S2A by RNA pull-down assays, HuR associated with the *p27* 5′UTR, 5′UTRb, and 3′UTR, while CUGBP1 only associated with the *p27* 5′UTR and 5′UTRb, in agreement with previous findings [15, 16]. NSun2 interacted with the *p27* 5′UTR and 5′UTRa, consistent with the results that NSun2 methylated *p27* 5′UTR and 5′UTRa (Fig. 2B). Although NSun2 was capable of interacting with the *p27* 3′UTR, this interaction did not lead to the methylation of *p27* 3′UTR (Fig. 2B). Furthermore, methylation by NSun2 did not influence the association of HuR or CUGBP1 with the *p27* mRNA (Supplemental Fig. S2B), and the association of NSun2 with *p27* mRNA did not influence the association of HuR and CUGBP1 with p27 mRNA or vice versa (Supplemental Fig. S2C-E). Moreover, knockdown of NSun2 increased the activities of reporters pGL3-5′UTR and pGL3-5′UTRa, but not those of pGL3-5′UTRb or pGL3-5′UTRΔ (Supplemental Fig. S2F). In contrast, knockdown of HuR or CUGBP1 increased the activities of reporters pGL3-5′UTR, pGL3-5′UTRb, and pGL3-5′UTRΔ, but not that of pGL3-5′UTRa (Supplemental Fig. S2F). These results suggest that the translational repression of p27 by NSun2-mediated mRNA methylation is independent of the effects elicited by HuR or CUGBP1.

### NSun2 regulation of p27 and CDK1 impacts upon replicative senescence

**Figure 4:**
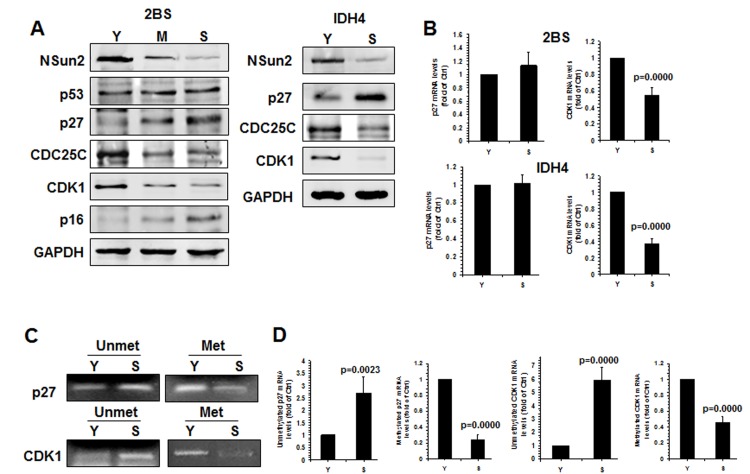
Inverse correlation between p27 levels and NSun2 levels in replicative senescence **(A)** *Left*, the levels of proteins NSun2, p53, p16, p27, CDC25C, CDK1, and GAPDH in early-passage (Proliferating ‘Young’, ‘Y’, ~PDL 28), middle-passage (middle, ~PDL 40), and late-passage (Senescent, ‘S’, ~PDL 55) human diploid fibroblasts (2BS) were assessed by Western blotting. *Right*, the levels of proteins NSun2, p27, and GAPDH in Proliferating (Y) and Senescent (S) IDH4 cells were analyzed by Western blot analysis. **(B)** RNA was prepared from young (Y, ~PDL 28) and senescent (S, ~PDL 55) 2BS cells as well as young (Y) and senescent (S) IDH4 cells and RT-qPCR analysis was performed to assess the levels of *p27* and *CDK1* mRNAs. **(C)** RNA described in Fig. 4B was subjected to methylation-specific PCR analysis to assess the methylation of *p27* and *CDK1* mRNA. Data are representative from 3 independent experiments. **(D)** The density of the methylation-specific PCR (% of Ctrl) in Fig. 4C relative to that of the mRNA levels (% of Ctrl) shown in Fig. 4B is shown. Data represent the means ± SD from 3 independent experiments; significance was analyzed by Student's *t* test.

Apart from p27, NSun2 was also found to regulate the expression of senescence-associated proteins p53, p16, CDK1, and CDC25C [26–28]. To further investigate whether NSun2 regulated the process of replicative senescence, we assessed the levels of proteins NSun2, p53, p16, CDK1, p27, CDC25C, and GAPDH in early-passage [proliferating, ‘Young’ (Y), ~PDL 28], middle-passage [middle (M), ~PDL 40], and late-passage [senescent (S), ~PDL 55] human diploid fibroblasts (2BS) by Western blot analysis. As shown in Fig. 4A (*left*), the levels of p16 and p27 increased with replicative replicative senescence, while the levels of p53 remained unchanged. However, the levels of proteins NSun2, CDK1, and CDC25C were reduced with replicative senescence. The inverse correlation between NSun2 and p27 levels was also observed in another model of senescence, proliferating and senescent IDH4 cells (Fig. 4A, *right*). In addition, the levels of *CDK1* mRNA decreased (by ~45% in 2BS cells, p=0.0000; by ~63% in IDH4 cells, p=0.0000) but *p27* mRNA levels remained unchanged during senescence in both cell types (Fig. 4B). Both endogenous p53 and p16 are constitutively expressed in IDH4 cells, regardless of whether they are maintained in a proliferative state or they are induced to undergo senescence (Fig. S3). Methylated RNA-specific PCR analysis revealed that the levels of methylated *CDK1* and *p27* mRNAs decreased in the process of replicative senescence (by ~76% for *p27* mRNA; by ~46% for *CDK1* mRNA) (Fig. 4C), while the levels of unmethylated *CDK1* and *p27* mRNAs increased in replicative senescence (by ~2.7 fold for *p27* mRNA, p=0.0023; by ~5.9 fold for *CDK1* mRNA). These results indicate that NSun2-mediated RNA methylation may be able to regulate the expression levels of CDK1, p27, and CDC25C, but not those of p53 and p16, as cells progress to senescence.

**Figure 5:**
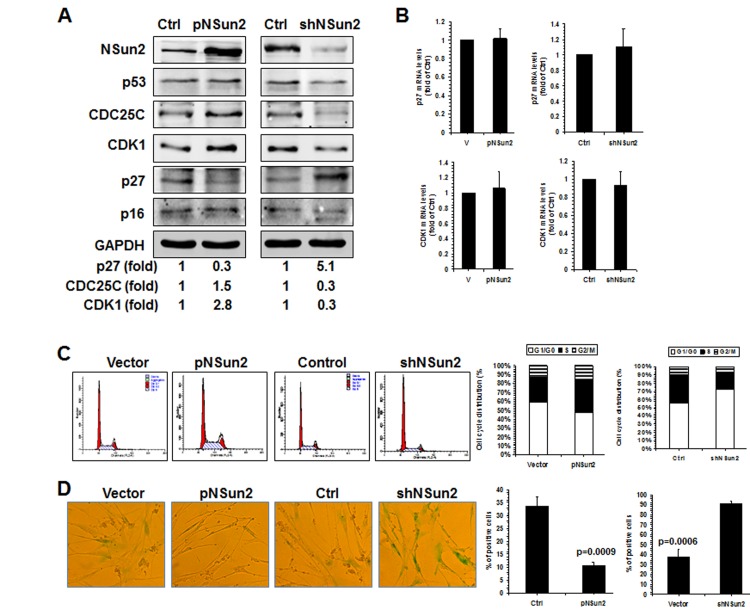
NSun2-p27 regulatory process impacts on the progression of replicative senescence **(A)** 2BS cells were stably infected with a lentivirus bearing a pHBLV-NSun2 vector (pNSun2) or a pHBLV-shNSun2 vector (shNSun2). Cell lysates were prepared and subjected to Western blot analysis to assess the levels of proteins NSun2, p53, CDC25C, p27, p16, CDK1, and GAPDH. **(B)** RNA prepared from cells described in Fig. 5A was subjected to RT-qPCR analysis to assess the levels of *p27* and *CDK1* mRNAs. Data represent the means ± SD from 3 independent experiments. **(C)** Cells described in Fig. 5A were analyzed for cell cycle distribution (*left*). The percentage of cells in each cell cycle compartment is presented (*right*). **(D)** Cells described in Fig. 5A were analyzed for the activity of SA-β-gal (*left*). The means ± SD from 3 independent experiments are presented; significance was analyzed by Student's *t* test (*right*).

To test this idea using a different approach, 2BS cells were stably infected with lentiviruses expressing either NSun2 or NSun2 shRNA. As shown in Fig. 5A, infection with the NSun2-expressing lentiviruses reduced the levels of p27 by ~70%, while infection with NSun2 shRNA-expressing lentiviruses increased the levels of p27 by ~5.1 fold. In contrast, the levels of proteins CDK1 and CDC25C increased in cells with overexpressed NSun2 (by ~2.8 fold for CDK1; by ~1.5 fold for CDC25C) but decreased in cells with silenced NSun2 (by ~70% for both CDK1 and CDC25C). However, the levels of proteins p53 and p16 increased moderately in cells with overexpressed NSun2 and decreased only moderately in cells with silenced NSun2 (Fig. 5A). These modest changes may be an indirect reflection of the fact that 2BS cells with overexpressed NSun2 or silenced NSun2 have a constitutively altered senescence phenotype, perhaps as a result of compensatory changes in response to the permanent alterations in NSun2 levels. In agreement with the findings in Fig. 1B and in previous studies [28], neither overexpression of NSun2 nor knockdown of NSun2 altered the levels of *p27* and *CDK1* mRNAs in a measurable manner (Fig. 5B). In addition, the levels of cellular methylated *p27* mRNA increased in cells with overexpressed NSun2 but decreased in cells with silenced NSun2 (Supplemental Fig. S4).

**Figure 6:**
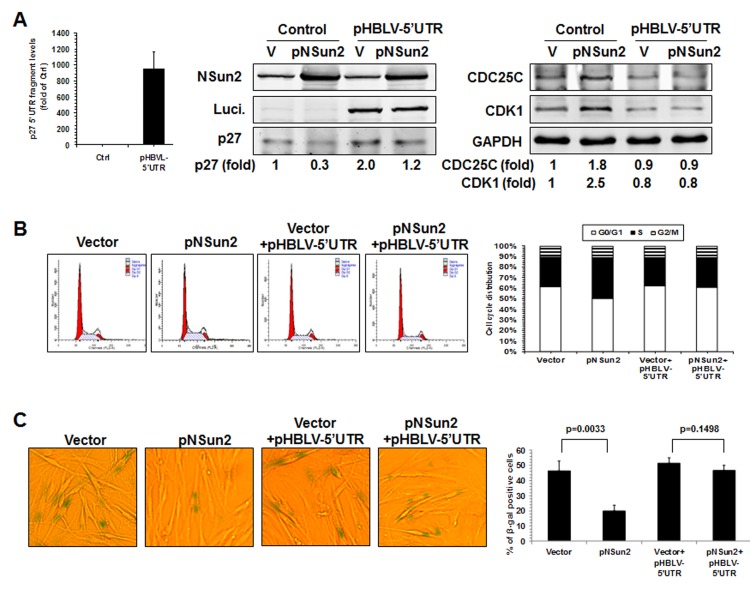
NSun2 delays replicative senescence by regulating p27, CDK1, and CDC25C **(A)** 2BS cells were stably infected with a lentivirus bearing a pHBLV-NSun2 vector (pNSun2) or with a control lentivirus (Vector), or stably co-infected with a lentivirus bearing a pHBLV-luc-5′UTR vector (Luc-5′UTR) or kept untreated (Control). Cell lysates were prepared and subjected to Western blot analysis to assess the levels of proteins NSun2, luciferase, p27, CDK1, CDC25C, and GAPDH. **(B)** The cells described in Fig. 6A were analyzed for cell cycle distribution (*left*) and the percentage of cells in each cell cycle compartment represented (*right*). **(C)** Cells described in Fig. 6A were used for analysis of SA-β-gal activity (*left*). The means ± SD from 3 independent experiments are presented and the significance was assessed by using Student's* t* test (*right*).

Finally, knockdown of NSun2 inhibited cell growth and increased the proportions of cells displaying the protein marker senescence-associated (SA)-β-galactosidase (~37.5% vs. ~91.0%), while overexpression of NSun2 promoted cell growth and reduced SA-β-galactosidase activity (~33.5% vs. ~10.6%). We next tested if overexpression of the* p27* 5′UTR fragment could function as a decoy fragment and rescue the effect of NSun2 overexpression in modulating p27, CDK1, and CDC25C levels, in promoting cell growth, and in delaying replicative senescence. As shown in Figure 6, overexpression of the *p27* 5′UTR fragment in 2BS cells by infecting 2BS cells with a lentivirus expressing luc-5′UTR (which increased *p27* 5′UTR levels by ~944 fold) rescued the effect of NSun2 overexpression in influencing the expression of p27, CDK1, and CDC25C (Figure 6A), in promoting DNA replication (progression through the S phase; Figure 6B), and in delaying replicative senescence (Control vs. pNSun2, ~46.4% vs. ~20.0%; control+pHBLV-5′UTR vs. pNSun2+pHBLV-5′UTR, ~51.5% vs. ~46.8%) (Figure 6C). Overexpression of the *p27* 5′UTR modestly increased the SA-β-galactosidase activity in control vector-transfected cells (~46.4% vs. ~51.5%), although the alteration was not statistically significant. In sum, the influence of NSun2 on the expression of p27, CDK1, and CDC25C has a robust impact upon replicative senescence.

## DISCUSSION

In response to oxidative stress, elevated NSun2 is responsible for the rise in p53 and p16 expression levels [26–27], suggesting that NSun2 is able to promote the process of oxidative stress-induced senescence. However, NSun2 does not appear to regulate replicative senescence by methylating *p53* and *p16* mRNAs, since reduced NSun2 in senescent HDFs does not result in reduced p53 and p16 levels (Fig. 4). Instead, the evidence obtained in the present study supports the view that NSun2-mediated mRNA methylation represses replicative senescence at least in part by lowering the translation of p27 and elevating the expression of CDK1 (Figs. 1–6). The NSun2-enhanced CDC25C expression, which is mediated by NSun2 regulation of miR125b [25], may also impact upon the process of replicative senescence (Figs. 4–6). It is possible that the role of NSun2 in replicative senescence is different from its role in oxidative stress-induced cellular senescence, since the levels of NSun2 protein decrease in replicative senescence (Fig. 4A) but increase in response to oxidative stress [26–27]. Accordingly, the mechanisms underlying the regulation of expression of certain genes (e.g., CDK1, p27, p16 or p53) during replicative senescence could be different from those operating during oxidative stress-induced senescence.

In previous studies, we demonstrated that methylation of 3′UTRs of different mRNAs by NSun2 increased the expression of p53, p16, E2F3, Bak1, ErbB2, and CDK1 [26–28]. In this study, methylation of p27 5′UTR by NSun2 repressed p27 translation (Figs. 1–3). Therefore, the regulatory effect of NSun2-mediated methylation may depend upon the location of methylation or the mRNAs targeted by NSun2. On the other hand, the association of NSun2 with *p27* 5′UTR is linked to the methylation of *p27* 5′UTR (Fig. 2B and Supplemental Fig. S2A). However, the association of NSun2 with *p27* 3′UTR (Supplemental Fig. S2A) did not result in methylation of *p27* 3′UTR (Fig. 2B) nor did it affect p27 expression levels (Fig. 3B). Therefore, whether the association of NSun2 with *p27* 3′UTR regulated the expression of p27 under other specific conditions or processes remains to be studied.

Translational regulation is critical for the elevation of p27, since *p27* mRNA levels do not change in the process of replicative senescence (Fig. 4B). Apart from NSun2, HuR and CUGBP1 also repress the translation of p27. Interestingly, HuR and CUGBP1 protein levels declined during replicative senescence [29–31]. Thus, it is plausible to postulate that HuR- and CUGBP1-mediated translational repression may be also involved in the regulation of p27 in replicative senescence, even though they appear to function independently of NSun2.

Proliferation arrest is a hallmark feature of cell senescence [1]. Therefore, genes involved in the regulation of the cell division cycle are extensively involved in the control of cellular senescence. Apart from the elevation in p27 levels, the reduction in CDK1 expression and CDK1 activity also contribute to growth arrest in cellular senescence [13,32]. Therefore, the repression of p27 translation and the elevation of CDK1 translation by NSun2-mediated mRNA methylation may cooperatively promote cell proliferation and delay the process of replicative senescence. In addition, although the role of CDC25C in replicative senescence remains untested, our results suggest that CDC25C may also inhibit growth during senescence (Figs. 4–6).

In general, genes highly expressed in senescent cells tend to show low expression levels in cancer, and vice versa. Indeed, induction of CDK1 has been linked to the growth of human colorectal cancer and acute myeloid leukemia [33–34]; the reduction of p27 has been liked to the genesis and progression of a broad array of human cancers, including cancers of the breast, colon, prostate, ovary, lung, stomach, and others [35–37]. The increase of NSun2 levels observed in a variety of human cancers, including those originating in the colon, esophagus, stomach, liver, oral cavity, pancreas, uterus, cervix, prostate, kidney, bladder, thyroid, breast, and skin [25, 38], may be part of a program of senescence repression involving reduced levels of p27. Besides cancer, p27 has also been implicated in aging-related diseases such as osteoporosis [39], atherosclerosis [40–41], and Alzheimer's disease [38]. It will be particularly important to investigate the possible involvement of the NSun2-p27 regulatory paradigm into these aging-related conditions.

## MATERIALS AND METHODS

### Cell culture, transfection, and SA-β-galactosidase activity

Human IDH4 fibroblasts were generously provided by J. W. Shay and cultured in Dulbecco's modified Eagle's medium (DMEM) (Invitrogen) supplemented with 10% fetal bovine serum, 100 units/ml penicillin, 100 μg/ml streptomycin, and dexamethasone (Dex, 1 μg/ml) for constitutive expression of SV40 large T antigen to suppress senescence and stimulate proliferation [29]. Proliferating (‘young’) and senescent IDH4 cells were obtained by incubating cells in charcoal-stripped serum (10%) in the presence (Young) or absence (Senescent) of Dexamethasone (Dex, 1 μg/ml) for 5 days. Early- [Proliferating (P), Young, ~28 population doublings (pdl)], middle- [(M), ~40 pdl], and late-passage [(S) Senescent, ~55 pdl] human diploid 2BS fibroblasts (National Institute of Biological Products, Beijing, China), and HeLa cells were cultured in DMEM supplemented with 10% fetal bovine serum, 100 units/ml penicillin, and 100 μg/ml streptomycin, at 37°C in 5% CO_2_. All plasmids were transfected using lipofectamine 2000 (Invitrogen) and collected 48 to 72 h after transfection for further analysis. SA-β-galactosidase activity (β-gal staining) was performed as described previously [42].

### Transcript preparation

cDNA was used as a template for PCR amplification of the p27 fragments. All 5′ primers contained the T7 promoter sequence (CCAAGCTTCTAATACGACTCACTATAGGGAGA). To prepare templates for the *p27* 5′UTR (positions 1-472), *p27* 5′UTR-A (positions 1-210), *p27* 5′UTR-B (positions 211-472), *p27* 5′UTRa1 (positions 1-140), *p27* 5′UTR a2 (positions 141-210), *p27* CR (positions 473-1069), and *p27* 3′UTR (positions 1070-2290), we used the following primer pairs: (T7) CTTCTTCGTCAGCCTCCC and CTTTCTCCCGGGTCTGCA for 5′UTR, (T7) CTTCTTCGTCAGCCTCCC and AGCGGAGAGGGTGGCAAAGCCC for 5′UTR-A, (T7) TGCCTGGTCCCCTCTCCTCT and CTTTCTCCCGGGTCTGCA for 5′UTR-B, (T7) CTTCTTCGTCAGCCTCCC and CTCTCCAAACCTTGCCGGCGTC for 5′UTRa1, (T7) CGGCTGGGTTCGCGGGAC and AGCGGAGAGGGTGGCAAAGCCC for 5′UTRa2, (T7) ATGTCAAACGTGCGAGTGTC and TTACGTTTGACGTCTTCTGAG for CR, (T7) ACAGCTCGAATTAAGAATATG and TAACAAAAGAGGGGAAAACCTATTC for 3′UTR. These PCR products were transcribed *in vitro* following the manufacturer's instructions (Thermo).

The *p27* 5′UTR mutants were prepared by overlapping PCR. The *p16* 3′UTR fragment was described previously [27].

### Western blot analysis

Monoclonal anti-CUGBP1, monoclonal anti-p53, monoclonal anti-p16, monoclonal anti-CDC25C, and monoclonal anti-HuR were from Santa Cruz Biotechnology. Polyclonal anti-NSun2 and monoclonal anti-GAPDH were from Abcam. After secondary antibody incubations, signals were detected by Odyssey Imaging System (Gene Company Limited) following the manufacturer's instruction and quantitated by densitometric analysis with Image J software.

### Knockdown of NSun2, HuR, and CUGBP1

To silence NSun2, HuR, or CUGBP1, cells were transfected with siRNAs (10 nmol/L each) targeting NSun2 (AGAUGUUAAGAUACUGUUGACCC), HuR (AAGAGGCAAUUACCAGUUUCAUU) or CUGBP1 (GAGCCAACCUGUUCAUCUAUU), or with a control siRNA (UUGUUCGAACGUG UCACGUTT) using RNAiMAX (Invitrogen). Unless otherwise indicated, cells were collected for analysis 48 h after transfection. All knockdown interventions caused less than 1% cell death (by FACS analysis, data not shown).

### RNA isolation and real-time qPCR analysis

Total cellular RNA was prepared using the RNeasy Mini Kit (Qiagen). For reverse-transcription (RT) followed by real-time, quantitative (q)PCR analysis of CDK1 and p27, we used following primer pairs: AAATGTGTGTAGGTCTCAC and ATGATTTAAG CCAACTCAAA for *CDK1* mRNA, and GGCTCCGGC TAACTCTGA and TCTTCTGTTCTGTTGGCTCTTT for *p27* mRNA.

### Constructs and reporter gene assays

For construction of the pGL3-derived reporter vectors bearing the *p27* 5′UTR and 5′UTR mutating C64 (C-G, 5′UTRΔ), the 5′UTR or 5′UTRΔ fragment was amplified by PCR by using primer pairs CCCAAGCTTGGGCTTCTTCGT CAGCCTCCC and CATGCCATGGCATGCTTTCT CCCGGGTCTGCA and inserted between the Hind III and Nco I sites of pGL3-promoter vector (Promega). For constructing the pGL3-derived reporter vectors bearing the *p27* 5′UTRa, 5′UTRb, CR, and 3′UTR, 5′UTRa, 5′UTRb, CR, and 3′UTR were amplified by PCR using primer pairs CCCAAG CTTCTTCGTCAGCCTCCC and CATGCCAGCGGA GAGGGTGGCAAAGCCC for 5′UTRa, CCCAAGTGCCTGGTCCCCTCTCCTCT and CATGCCCTTTCTCCCGGGTCTGCA for 5′UTRb, CCCAAGCTGTCAAACGTGCGAGTGTC and CATGCCTTACGTTTGACGTCTTCTGAG for CR, and CCCAAGACAGCTCGAATTAAGAATATG and CATGCCTAACAAAAGAGGGGAAAACCTATTC for 3′UTR. Each of these fragments was inserted between HindIII and NcoI sites of the pGL3-promoter vector (Promega). The pcDNA 3.1 vector expressing NSun2 was described previously [27].

For reporter gene assays, each of the pGL3-derived vectors was co-transfected with pRL-CMV vector by Lipofectamine 2000 (Invitrogen). Forty eight hours after transfection, cell lysates were collected and the firefly and renilla luciferase activities were measured with a double luciferase assay system (Promega) following the manufacturer's instructions. All firefly luciferase measurements were normalized to renilla luciferase measurements from the same sample.

### Lentiviruses and stable expression of ectopic genes

For the construction of lentivirus vectors expressing NSun2 (pHBLV-NSun2), the coding region of NSun2 was amplified by PCR by using primer pairs CGGAATTCATGGGCGGCGGTCGCGGGGT and CGGGATCCGGTCACCGGGGTGGATGGACC and inserted between EcoRI and BamHI sites of pHBLV-CMVIE-IRES-Puro vector (HANBIO Biotechnology Co., Ltd, Shanghai, China). For construction of the lentivirus vector expressing Luc-5′UTR (pHBLV-luc-5′UTR), the Luc-5′UTR fragment was amplified from the pGL3-5′UTR reporter by PCR by using primers CCGGAATTCTTCTTCGTCAGCCTCCC and CGGGATCCCGTTACACGGCGATCTTTCCGCCCT and inserted between EcoRI and BamHI sites of pHBLV-CMVIE-IRES-Puro vector. For construction of the lentivirus vector expressing NSun2 shRNA (pHBLV-shNSun2), complementary fragments GATCCGCGGCCTCATCATAAGATCTTAGATATTCAAGAGATATCTAAGATCTTATGATGAGGCCGTTTTTTC and AATTGAAAAAACGGCCTCATCATA AGATCTTAGATATCTCTTGAATATCTAAGATCTTATGATGAGGCCGCG were annealed and inserted between the BamHI and EcoRI sites of pHBLV-U6-Zsgreen-puro vector (HANBIO Biotechnology Co., Ltd, Shanghai, China). Viruses were packaged in 293T cells following the instructions from the manufacturer (HANBIO Biotechnology Co., Ltd, Shanghai, China). Stably transfected cells (2BS) were selected in the presence of puromycin (0.5 μg/ml) for 5 days.

### Preparation of polysomal fractions

A total of 20 million cells were incubated for 15 min with 100 mg/ml cycloheximide, and total lysates (500 μl) were layered onto a cushion of 30% sucrose in ice-cold buffer containing 20 mM HEPES (pH 7.4), 50 mM potassium acetate, 5 mM magnesium acetate, 1 mM dithiothreitol, 1 unit of RNasin per μl, 1 μg of leupeptin per ml, 1 μg of aprotinin per ml, and 0.5 mM phenylmethylsulfonyl fluoride. After centrifugation (Beckman SW40; 100,000 × *g* for 2 h, 4°C), RNA from the supernatant (nonpolysomal fraction) and the pellet (polysomal fraction) was prepared and used for RT-qPCR analysis.

*In vitro* methylation assays. For *in vitro* methylation assay, His-tagged NSun2 was prepared as described previously [27]. Briefly, reaction mixtures (50 μl) containing 0.2 nM His-NSun2, 0.01 nM RNA, and 1 μCi of ^3^H-labeled S-adenosyl-L-methionine (SAM, Amersham Bioscience) in reaction buffer (5 mM Tris HCL[pH 7.5], 5 mM EDTA, 10% glycerol, 1.5 mM dithiothreitol, 5 mM MgCl_2_) supplemented with inhibitors (leupeptin [1 μg/ml], aprotinin [1 μg/ml], 0.5 mM phenyl-methylsulfonyl fluoride, and RNasin [5 U/μl]), incubated for 45 min at 37°C, as described [27]. E. coli tRNA (0.01 nM, Sigma) and p16 CR (0.01 nM) served as a positive and negative controls, respectively. Unincorporated ^3^H SAM was removed by using Qiaquick Spin Columns (Qiagen) and incorporated radioactivity was measured by liquid scintillation counting. The non-isotopic methylated RNA fragments were prepared using cold SAM (Sigma) and *in vitro*-transcribed RNA fragments under similar conditions.

### Bisulfate RNA sequencing

This method could identify the methylation site (m5C) within an RNA fragment shorter than 150 nt. Briefly, fragment 5′UTRa1 (positions 1-140), which was methylated by NSun2, was amplified by using primers (T7) CTTCTTCGTCAGCCTCCCTT and CCTCCATTCC CAACAAACCTTGCCGGCGTCGGAGTCGCAGAGCCGTGA.

This fragment (1 μg) was transcribed *in vitro* and methylated by using non-isotopic SAM. Samples then were dissolved in 10 μl of RNase-free water and mixed with 42.5 μl 5 M sodium bisulfate (Epitect) and 17.5 μl DNA protection buffer (Epitect), incubated in 70°C for 5 minutes and 60°C for 60 min, repeating for 3-5 cycles. After desalting using Micro Bio-spin6 columns, samples were de-sulfonated by 1 M Tris (pH 9.0, 1/1, V/V) at 37°C for 1 h, followed by ethanol precipitation. The bisulfate-converted fragments (0.2 μg) were reverse-transcribed by RevertAid First Strand cDNA Synthesis Kit (Thermo) using primer GTCGTATCCAGTGCAGGGTCCGAGGTATTCGCACTGGATACGACCCTCCATTCCCAACAAAC. The reverse-transcribed products (cDNA) were amplified by PCR by using primers GGGAGATTTTTTTGTTAGT and GCAGGGTCCGAGGTATTC. The PCR products were inserted into the pGEM-T Easy Vector System (Promega). The plasmids purified from single clones were sequenced. The sequences were aligned with the corresponding *p27* mRNA sequence and the cytosines retained were considered to be methylated.

### Methylation-specific RT-PCR

Two micrograms (2 μg) of cellular RNA or 1 μg of *in vitro*-methylated RNA fragment was bisulfite-converted as described in ‘bisulfate RNA sequencing’. The converted *p27* 5′UTR was reverse-transcribed by using primer GTCGTATCCAGTGCAGGGTCCGAGGTATTCGCACTGGATACGACCCAACCACTCTCCAAACCTT. For methylation-specific *p27* mRNA RT-PCR analysis, primer pairs TAATAAGGTTAAAAAAGGGGGTTTG TTTGGGC and GCAGGGTCCGAGGTATTC (for methylated fragment) as well as AAAAAAGGGGG TTTGTTTTAAT and GCAGGGTCCGAGGTATTC (for unmethylated fragment) were used.

### *In vitro* translation assays

For *in vitro* translation assays, a cell-free translation system (Promega) in rabbit reticulocyte lysate (RL) was used. Luciferase (Luc) transcript was* in vitro* transcribed from pGL3 by using primer pairs (T7) ATGGAAGACGCCAAAAA CAT and TTTTTTTTTTTTTTTTTTTTTTTTTTTTT TACACGGCGATCTTTCCGCCCT. Luc-5′UTR and Luc-5′UTRΔ transcripts were transcribed *in vitro* from pGL3-5′UTR and pGL3-5′UTRΔ reporters by using primer pairs (T7) CTTCTTCGTCAGCCTCCCTT and TTTTTTTTTTTTTTTTTTTTTTTTTTTTTTACACG CGATCTTTCCGCCCT. Transcripts (0.01 nM) were either methylated by NSun2 or kept untreated. The methylated and non-methylated transcripts were used for *in vitro* translation assays. The translation efficiency was determined by measuring the activity of firefly luciferase.

## SUPPLEMENTAL METHODS FIGURES


