# Supplementary material for: NSun2 delays replicative senescence by repressing p27 (KIP1) translation and elevating CDK1 translation
Source: Aging (Albany NY). 2015 Dec 14;7(12):1143–55. doi: 10.18632/aging.100860 (PMC4712338; doi:10.18632/aging.100860)
Supplement: Supplementary file 1 [file aging-07-1143-s001.pdf]

SUPPLEMENTAL METHODS

<u>LC-MS analysis.</u> *In vitro*methylated RNA fragments (1µg) were digested by nuclease P1 (Sigma) and alkaline phosphatase (Thermo). The formation of m5C or m6A was analyzed by HPLC-MS analysis at Tsinghua University Mass Spectrum Center (Beijing, China).

<u>RNA pullpdown assays.</u> For biotin pull-down assays, PCR-amplified DNA was used as template to transcribe biotinylated RNA by using T7 RNA polymerase in the presence of biotin-UTP. One microgram of purified biotinylated transcripts were incubated with 100 µg of cytoplasmic extracts for 30 min at room temperature. Complexes were isolated with paramagnetic streptavidin-conjugated Dynabeads (Dynal, Oslo), and the pull-down material was analyzed by Western blotting.

SUPPLEMENTAL FIGURES

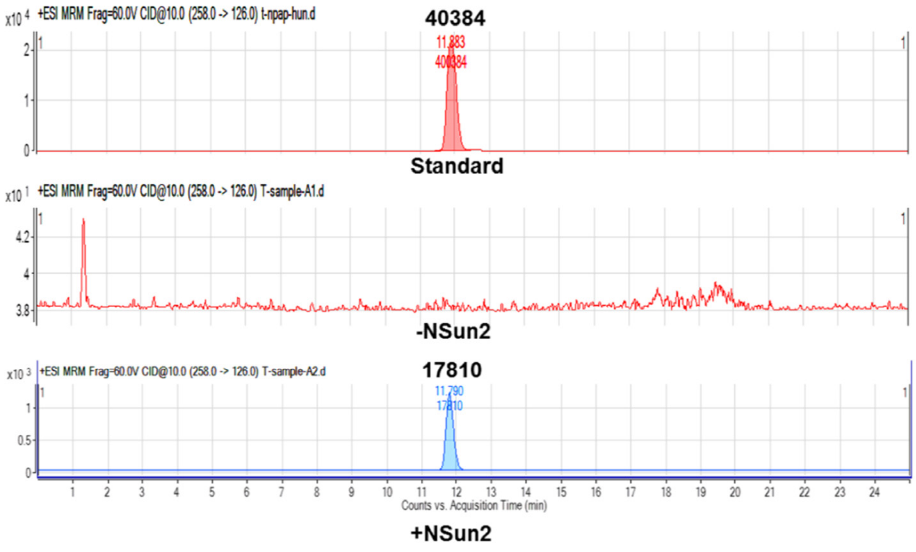

Supplemental Figure S1. *In vitro* methylated (+NSun2) or unmethylated (-NSun2) *5'UTRa 1*RNA was subjected to HPLC-MS analysis to determine the formation of m5C.The peak value of m5C was indicated on the top of the peaks.

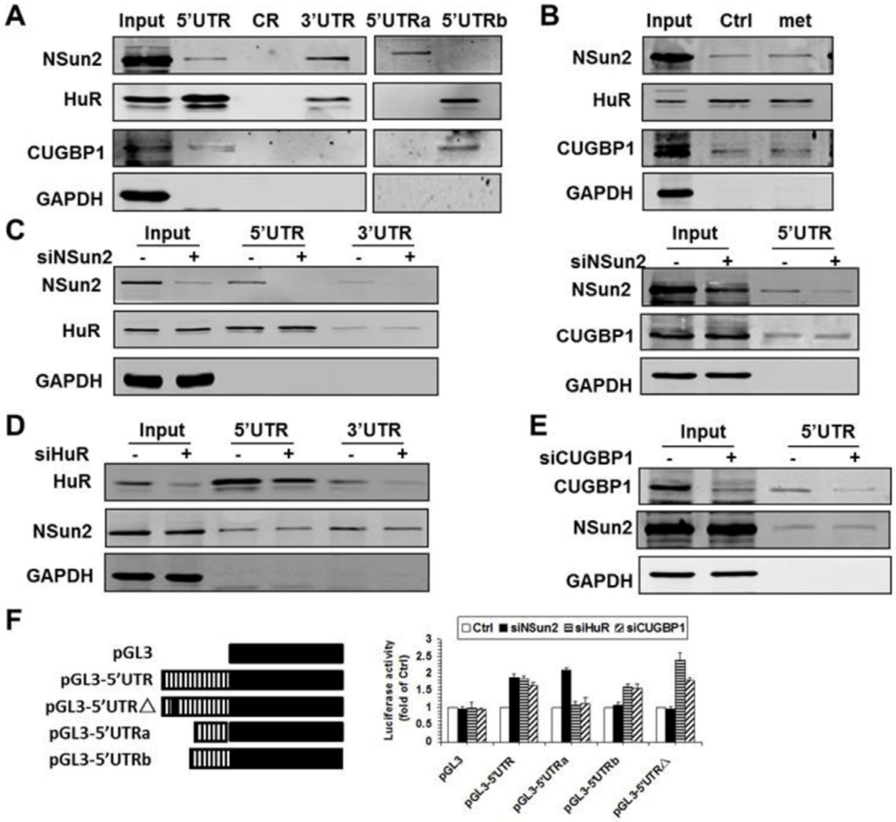

Supplemental Figure S2. Translational repression of p27 by NSun2 is independent of HuR or CUBP1. (A) RNA pull-down assays using biotinylated *p27*5'UTR, CR, 3'UTR, 5'UTRa, and 5'UTRb fragments and HeLa cell lysates. The presence of NSun2, HuR, and CUGBP1 in the pulldown materials was tested by Western blot analysis. A 10-µg aliquot of whole-cell lysates (Input) and bound GAPDH were included. **(B)** Biotinylated *p27*5'UTR fragment was methylated *in vitro* (Met) by NSun2 or left unmethylated (Unmet). The methylated and unmethylated 5'UTR fragments then were subjected to RNA pulldown assays to assess the association of *p27* 5'UTR with NSun2, HuR, and CUGBP1, as described in Figure S2A.**(C)**HeLa cells were transfected with a siRNA targeting NSun2; 48 h later, cell lysates were prepared and subjected to RNA pulldown analysis as described in Figure S2A to assess the presence of HuR in the pulldown materials of p27 5'UTR and 3'UTR fragments (*left*) as well as the presence of CUGBP1 in the p27 5'UTRfragment (*right*). **(D, E)** HeLa cells were transfected with a siRNA targeting HuR (D) orCUGBP1 (E); 48 h later, cell lysates were prepared and subjected to RNA pulldown assays as described in Figure S2A to assess the association of NSun2 with the *p27* 5'UTR fragment.**(F)**HeLa cells were transfected with each of the pGL3-derived reporter vectors (*left*, Schematic)together with a pRL-CMV control reporter. Twenty-four h later, cells were further transfected with siRNA stargeting NSun2, HuR, or CUGBP1 and cultured for additional 48 h. Firefly luciferase activity was assayed relative to Renilla luciferase activity. Data represent the means ± SEM from 3 independent experiments.

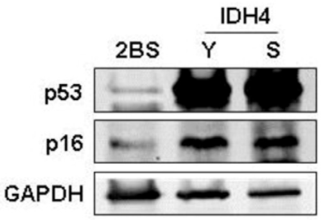

Supplemental Figure S3. Constitutive expression of p53 and p16 in proliferating and senescent IDH4 cells. The levels of p53, p16, and GAPDH in proliferating (Y) and senescent (S) IDH4 cells were analyzed by Western blot analysis. The levels of proteins p53, p16, and GAPDH in mid-passage human diploid fibroblast (2BS, ~PDL 35) served as controls.

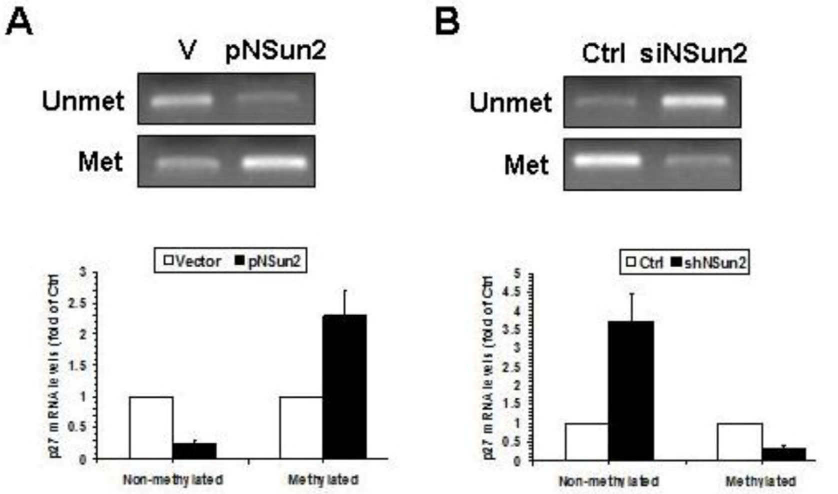

Supplemental Figure S4. Modulating NSun2 levels in 2BS cells alters the cellular methylation levels of *p27*mRNA. **(A, B)** RNA isolated from cells described in Fig. 5A were subjected to methylation-specific PCR analysis to assess the levels of unmethylated and methylated *p27*mRNA. Data represent the means ± SEM from 3 independent experiments.
